# All-Optical Graphene Oxide Humidity Sensors

**DOI:** 10.3390/s141224329

**Published:** 2014-12-17

**Authors:** Weng Hong Lim, Yuen Kiat Yap, Wu Yi Chong, Harith Ahmad

**Affiliations:** 1 Photonics Research Centre, University of Malaya, Kuala Lumpur 50603, Malaysia; E-Mails: wghglim@hotmail.com (W.H.L.); yuenkiat@siswa.um.edu.my (Y.K.Y.); harith@um.edu.my (H.A.); 2 Department of Physics, Faculty of Science, University of Malaya, Kuala Lumpur 50603, Malaysia; 3 Centre for Foundation Studies, SEGi University, Kota Damansara 47810, Selangor, Malaysia

**Keywords:** graphene oxide, humidity sensing, optical sensing

## Abstract

The optical characteristics of graphene oxide (GO) were explored to design and fabricate a GO-based optical humidity sensor. GO film was coated onto a SU8 polymer channel waveguide using the drop-casting technique. The proposed sensor shows a high TE-mode absorption at 1550 nm. Due to the dependence of the dielectric properties of the GO film on water content, this high TE-mode absorption decreases when the ambient relative humidity increases. The proposed sensor shows a rapid response (<1 s) to periodically interrupted humid air flow. The transmission of the proposed sensor shows a linear response of 0.553 dB/% RH in the range of 60% to 100% RH.

## 1. Introduction

Aside from the basic needs of high sensitivity and wide detection range additional demands for fast response and short recovery time exist for humidity sensors to meet industry requirements. Consequently, research on humidity-sensitive nanomaterials such as silicon nanostructures, ceramic nanomaterials, semiconductor nanoparticles and metal oxide nanowires have attracted much attention due to its high surface to volume ratio [1,2]. Lately, monolayers or a few layers of graphene followed by graphene oxide (GO) have received increasing attention due to their inherent electrical and mechanical properties, holding great potential for ultrasensitive sensor applications [1,3]. Schedin demonstrated that graphene has single molecule detection sensitivity due to its low thermal noise properties [4]. On the other hand, GO has many oxygen functional groups bonded in its two-dimensional network of sp^2^ and sp^3^ hybridized carbon atoms arranged in a honeycomb structure. These functional groups include hydroxyl, epoxy and carboxylic acid moieties [1,3]. The sp^3^ hybridized carbon atoms turn GO into an insulator by decreasing its conductivity, and enhance its hydrophilic properties, [3,5,6]. Besides, oxygen functional groups in GO also allow fast water permeation within the GO layers as reported by Nair [7], which enables fast water molecule diffusion (in and out) of GO during humidity changes.

Capacitive-based sensors having high response time, low temperature dependence, low hysteresis, low drift with good performance in harsh environment are desirable in various applications [8–14]. During the past five years, many research journals have reported on the favorable properties of GO in various sensing applications with different designs such as direct measurement of capacitance between two parallel plates or in a slightly more complex CMOS architecture where the GO acts as insulating layers in the capacitor [1,3–6,15–18]. The overall working mechanisms of these works are mainly based on the measurement of GO conductivity (or more precisely, capacitance) in DC or low AC regimes (up to a few kHz), with and without the measurands. To the best of our knowledge, no work has been reported on the optical frequency response of GO to relative humidity changes. Optical sensing with fibre optics technology is a proven method with its own notable advances [19–22], especially for long distance monitoring of underground pipe networks, as compared to electrical sensing which can suffer from relatively high transmission losses as well as electromagnetic interference noise. Hence, in this work we explore the application of GO in optical sensing by studying the characteristics of its optical response to relative humidity changes.

## 2. Device Fabrication and Experiment

**Figure 1:**
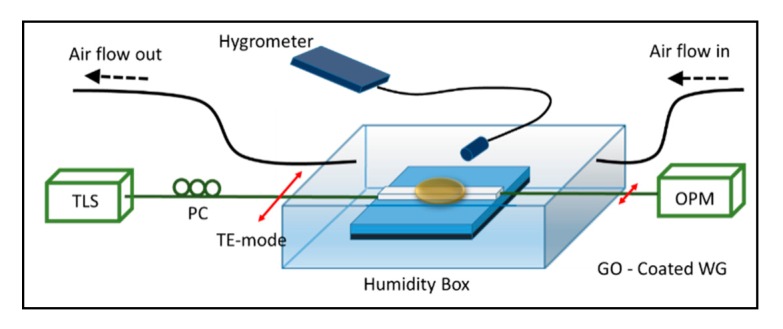
Schematic diagram of the proposed sensor—A GO-coated SU8 polymer waveguide in a humidity measurement setup.

The experimental setup of the humidity sensor is shown in Figure 1. The polymer-based waveguide sensor was fabricated on a silicon substrate coated with BCB 4024-40 polymer as the underclad layer. BCB with a thickness of 6.1 μm and refractive index of 1.5538 measured at 1550 nm using a Sairon Technology SPA-4000 prism coupler was spin-coated on the silicon substrate and thermally cured at 250 °C for 1 h. Then, SU8 polymer with a refractive index of 1.566 measured at 1550 nm was spin-coated onto the BCB undercladding layer and patterned using a contact photolithography technique. The fabricated SU8 channel waveguide has a cross section of 5 μm in height and 10 μm in width. The sample then underwent a second thermal curing process at 80 °C for 4 h. The cured sample was then cut into chips about 1 cm in length and carefully bonded with optical fibre interconnects using NOA-65 UV-sensitive resin for optical signal coupling purposes.

On the other hand, GO solution was prepared by an improved version of Hummer's method [23]. The GO solution in de-ionized water as the solvent has a concentration of about 1 μg/μL. Three drops of 1 μL of GO solutions were applied directly onto the polymer waveguides via a drop-casting method. Drop-casting of GO solutions to form a uniform GO film was demonstrated by Sun [24]. Using the multiple-drop-casting technique with small volume instead of larger volume per-drop helps to achieve a smaller coated area while obtaining a thicker GO film. The sample was then allowed to dry under ambient conditions. The resulting GO coating has a diameter of 2 mm and its average thickness was measured using a Dektak D150 surface profiler and found to be about 0.87 μm. It is worth noting that the adhesion of the GO film is relatively strong whereby it can only be removed or damaged by a strong physical scratch. The uncoated SU8 waveguide section was covered using NOA-65 UV-sensitive resin to ensure that only the GO film is exposed to the ambient environment. The optical polarization state supported by the GO coated waveguide was measured using a polarimeter (Thorlabs PAX 5710, Newton, NJ, USA). It was found that the TE-polarized light is suppressed by the GO-coated waveguide to more than −20 dB with reference to TM-polarized light. Suppression of the TE-mode is due to absorption of electric field by the GO layers, which was reported in [25].

A gas tube providing controlled humidity vapour was placed about 1 cm above the GO-coated waveguide (sensing region) of the proposed sensor. Humid nitrogen gas was produced by bubbling dry nitrogen gas through water before flowing out from the gas tube. The response time of the proposed sensor to humid air (∼90% RH) was measured using a photodiode and by placing a mechanical shutter in between the gas source and the sensing region to control the exposure intervals. Measurement of the change in transmitted power to different relative humidity values was carried out by placing the proposed sensor in a humidity control box. Relative humidity in the box was controlled by mixing dry nitrogen gas and humid nitrogen gas with different mixing flow rates before flowing into the box. Before each optical measurement, the relative humidity of the gas flow or inside the humidity control box was measured using a HI 8562 hygrometer from Hanna Instruments. A long working distance optical microscope (Nikon SMZ1000, Kuala Lumpur, Malaysia) was used to monitor the sensing region during the experiment. Throughout the optical measurement, the polarization of the incident light was set to be TE-polarized using a polarization controller (PC). Extra care in the handling of the launch fibre was taken to ensure that the linear polarization state of the incident light was not scrambled by minor disturbances on the fibre. Also, the polarization dependent loss between TE- and TM-polarized modes of the incident light for another uncoated polymer waveguide was measured and found to be lower than 0.5 dB, limited only by the performance of the PC.

## 3. Results and Discussion

**Figure 2:**
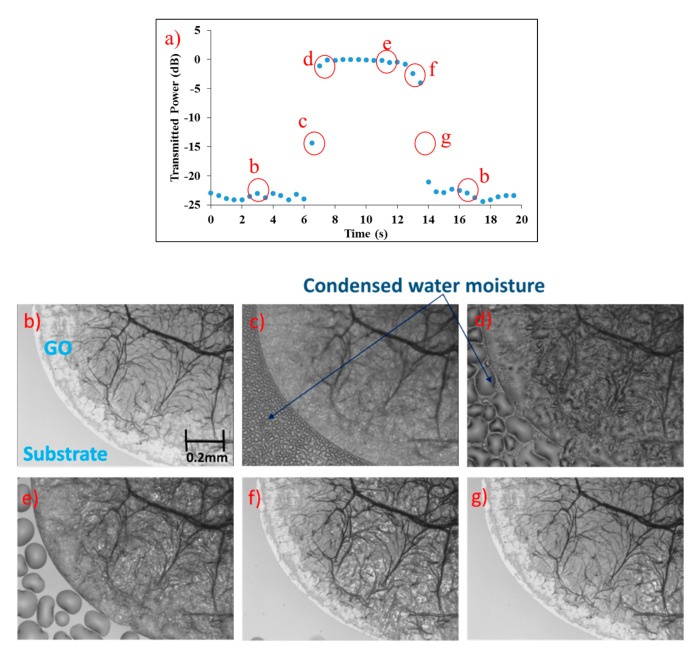
(**a**) Change in transmitted power over time when humid air was introduced onto the GO film, from the 6th s to 9th second, and corresponding optical micrographs of the GO film at the marked times: (**b**) before humid air was introduced; (**c**) the beginning of the flow of humid air at the 6th second; (**d**) the end of the humid air flow; (**e**) first drying stage of the GO film with water droplets on substrate; (**f**) second drying stage, where water droplets on substrate and GO film has evaporated; and (**g**) final stage of drying where water content in the GO film recedes rapidly from the edge to the centre.

Figure 2a shows the response of the proposed sensor under ambient conditions (∼50% RH) to a breeze of humid nitrogen gas (100% RH) over time. The breeze was introduced directly onto the sensing region of the proposed sensor, and sustained from the 6th to 9th second. The transmitted power increased instantaneously to its maximum (equivalent to the transmitted power of the TM-polarized mode). It remained at maximum for 6 s before returning to its initial minimum transmitted power level, starting at the 13th second till the 14th second. Optical micrographs of the physical appearance of sensing region at different stages in Figure 2a are shown in Figure 2b–g.

Prior to the introduction of humid air (up to the 6th second), no water condensation was observed on either the GO film or the substrate, as shown in Figure 2b. When humid air was first introduced at approximately the 6th second, corresponding to the increase in transmitted power, small water droplets were formed on the substrate surface and a change in the GO film brightness contrast was observed (Figure 2c). This indicates fast water permeation into the GO film, which reduces the propagation loss of TE-polarised light induced by the GO film. Sustained humid air flow onto the sensor for a further 3 s created larger water droplets on the substrate surface (Figure 2d). A layer of water was also observable on the GO film but this did not affect the transmitted power. When the humid air flow was stopped, two drying stages were observed. The first drying stage involves the drying of water moisture droplets on the substrate and GO film surface (Figure 2e—9th–13th second). At this stage, water still remained in the GO film, indicated by the difference in brightness contrast of the GO film between Figure 2b and Figure 2e and the constant transmitted power of the proposed sensor. The second drying stage (13th to 14th second) involves the drying or reduction of water content in the GO film from the edge towards the centre of the film, observed as the restoration of brightness contrast from the edge towards the centre of the GO film (Figure 2f and Figure 2g). This corresponds to the decrease of transmitted power to its initial value, and the GO film looked similar to Figure 2b after the 14th second. The transmitted power did not decrease immediately as anticipated at the 9th second due to the fact that the GO film was saturated with water, where complete evaporation involves first the evaporation of surface water, followed by water content inside the GO film. The drying process will cause hysteresis if the proposed sensor is subjected to humid air with 100% RH, which will result in condensation of water on the GO film. Nevertheless, these observations showed that humid air can easily permeate into the GO film and results in a change of the transmitted power of the proposed sensor. The experiment was repeated five times and the proposed sensor showed similar responses each time.

**Figure 3:**
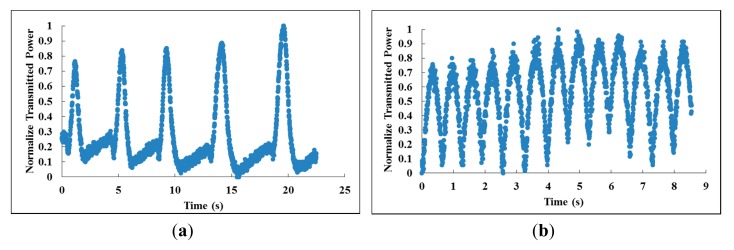
Changes in the normalized transmitted power when humid air (100 RH%) was introduced onto the proposed sensor periodically at (**a**) 5.0 s intervals and (**b**) 0.67 s intervals.

To study the response time of the proposed sensor, the output of the sensor was connected to a photodiode and the transmitted power was observed using an oscilloscope, and the results are shown in Figure 3. Humid air flow onto the sensing region was controlled using a mechanical shutter with flow intervals of 5 s and a flow time of 1 s. The proposed sensor exhibits a fast response to the presence and absence of humid air flow as shown in Figure 3a. The gradual increase in maximum transmitted power for subsequent breeze of humid air flow indicates that the GO film was not saturated with water during the first few breezes of humid air flow. When the flow interval was reduced to ∼0.67 s, the proposed sensor is still able to provide a distinction between the presence and absence of humid air flow, as shown in Figure 3b. Shorter flow intervals will result in the saturation of the GO film with water and the transmitted power of the proposed sensor will eventually remain at the maximum after a few flow intervals. This is also verified by the visual inspection of the GO film using microscope.

**Figure 4:**
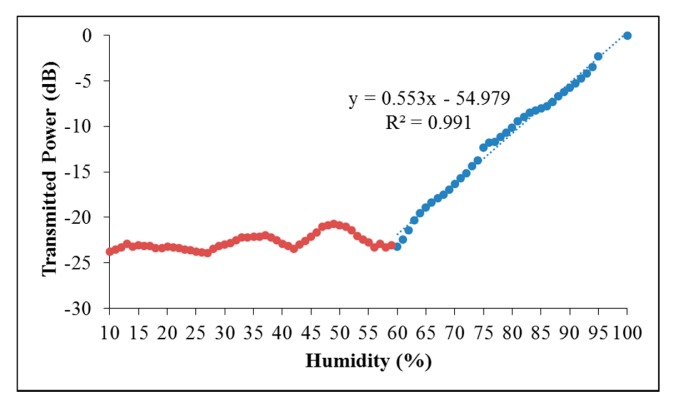
Linear response to humidity in the range of 60% RH to 100% RH.

Figure 4 shows the transmitted power of the proposed sensor at different humidity levels. Basically it shows two characteristics. For humidity levels below 60% RH, the transmitted power of the proposed sensor remained low and did not show any changes while a linear response was observed for humidity level between 60 to 100% RH with the transmitted power increased linearly from −23 dB to 0 dB respectively. Due to range limitation of the calibration hygrometer, relative humidity from 95% to 99% RH was not measured, while 100% RH was determined by the observation of water condensation on the surface of the sensing region. The response of the proposed sensor is calculated to be 0.553 dB/% RH in humidity range between 60 to 100% RH.

The polarization state of the transmitted light was measured when the proposed sensor was exposed to a relative humidity of 100% RH, and was TE-polarized. In fact, rotating the PC only rotates the polarization state and does not affect the transmitted power. The water saturated GO film seems to lose its polarization capability and becomes transparent to both TE- and TM-polarized light. There are reports showing that, at high relative humidity, water molecules can easily permeate in and out of the GO film of up to 10 μm in thickness [7]. The permeated water molecules readily interact with the functional groups of the individual GO layer [15,26,27]. This interaction changes the dielectric properties of the GO film and results in further widening of the GO layers bandgap [6,16,28], which corresponds to a decrease in conductivity. Henceforth, the propagation loss of the TE-polarized light in the GO film is reduced, resulting in the increase of the transmission of TE-polarized light. When the relative humidity surrounding the sensing was increased, the amount of water permeated into the GO film will increase in order to achieve equilibrium with the surrounding air. The result is the increase in transmitted power of the proposed sensor with the increase of relative humidity. Below 60% RH (plotted as brown coloured markers in Figure 4), the proposed sensors showed a flat response, which indicates that water permeation into the GO film is not significant for relative humidity below 60% RH. This phenomenon is consistent with [7], where the increase of water permeation rate through GO film with increasing relative humidity only becomes significant at high relative humidity.

## 4. Conclusions

The humidity sensing ability of a GO-coated optical waveguide has been studied. The optical transmission characteristics of the proposed sensor are found to be affected by the relative humidity of its surroundings. The increase in transmitted power at higher humidity is due to a change in the dielectric properties of the GO film where the conductivity of the GO film is reduced in the presence of water molecules. The proposed sensor exhibits fast response of less than 1 s to humid air breezes. In addition, it shows a good linear response of 0.553 dB/% RH in the humidity range from 60% to 100% RH. The all optical nature of the proposed sensor enables its application in long distance humidity sensing with fast response.
